# Dorsomedial prefrontal cortex activity predicts the accuracy in estimating others' preferences

**DOI:** 10.3389/fnhum.2013.00686

**Published:** 2013-11-26

**Authors:** Pyungwon Kang, Jongbin Lee, Sunhae Sul, Hackjin Kim

**Affiliations:** ^1^Laboratory of Social and Decision Neuroscience, Korea UniversitySeoul, South Korea; ^2^Department of Brain and Cognitive Engineering, Korea UniversitySeoul, South Korea; ^3^Department of Psychology, Korea UniversitySeoul, South Korea

**Keywords:** preference estimation, dorsomedial prefrontal cortex, temporoparietal junction, posterior cingulate cortex/precuneus, thin-slice judgment, theory of mind

## Abstract

The ability to accurately estimate another person's preferences is crucial for a successful social life. In daily interactions, we often do this on the basis of minimal information. The aims of the present study were (a) to examine whether people can accurately judge others based only on a brief exposure to their appearances, and (b) to reveal the underlying neural mechanisms with functional magnetic resonance imaging (fMRI). Participants were asked to make guesses about unfamiliar target individuals' preferences for various items after looking at their faces for 3 s. The behavioral results showed that participants estimated others' preferences above chance level. The fMRI data revealed that higher accuracy in preference estimation was associated with greater activity in the dorsomedial prefrontal cortex (DMPFC) when participants were guessing the targets' preferences relative to thinking about their own preferences. These findings suggest that accurate estimations of others' preferences may require increased activity in the DMPFC. A functional connectivity analysis revealed that higher accuracy in preference estimation was related to increased functional connectivity between the DMPFC and the brain regions that are known to be involved in theory of mind processing, such as the temporoparietal junction (TPJ) and the posterior cingulate cortex (PCC)/precuneus, during correct vs. incorrect guessing trials. On the contrary, the tendency to refer to self-preferences when estimating others' preference was related to greater activity in the ventromedial prefrontal cortex. These findings imply that the DMPFC may be a core region in estimating the preferences of others and that higher accuracy may require stronger communication between the DMPFC and the TPJ and PCC/precuneus, part of a neural network known to be engaged in mentalizing.

## Introduction

We often need to infer another person's preferences based on very limited information in daily life. For example, we choose a restaurant for dinner with an invited speaker whom we have never met before, make a plan for a first date, prepare a small gift for a new business partner, or rely on our intuitive feelings about customers to see through their preferences. Although estimating the preferences of others frequently occurs without prior knowledge, most studies on this topic have focused on how people utilize known information to estimate preferences (Hoch, 1988; West, 1996; Lerouge and Warlop, 2006). Only recently, North and colleagues have shown that people can estimate the preferences of others based on shortly presented subtle and non-communicative facial expressions (North et al., 2010). The present study centered on the ability to accurately estimate another person's preferences on the basis of minimal information.

The ability to infer about others quickly and act accordingly is important for leading a successful social life, and this kind of intuitive social inference has been well documented in social psychology literature (Funder and Harris, 1986; Ambady and Rosenthal, 1992; Zaki and Ochsner, 2011). It is known that people can infer various types of information, such as personality (Berry, 1991; Gosling et al., 2002), trustworthiness (Engell et al., 2007; Van't Wout and Sanfey, 2008), competence (Todorov et al., 2005), altruism (Fetchenhauer et al., 2010), socioeconomic status (Kraus and Keltner, 2009), sexual orientation (Rule et al., 2009; Freeman et al., 2010a), violence of sexual offenders (Stillman et al., 2010), as well as preferences (North et al., 2010), on the basis of a brief (usually ranging from 2 s to 5 min) exposure to facial appearance or to an excerpt of behavior. Ambady and colleagues have emphasized the adaptive function of accurately judging others based on minimal information (Ambady et al., 1995) and have suggested that this ability reflects the interpersonal sensitivity of an individual (Ambady et al., 2001). Despite a large body of behavioral evidence, only a few neuroimaging studies have investigated the neural mechanisms underlying the accuracy of personal traits that are inferred from facial appearances (Spezio et al., 2008; Rule et al., 2010, 2011), and, most importantly, no studies have been conducted on the accuracy of estimating the preferences of others.

Of most relevance to the current work are recent studies on the role of the dorsomedial prefrontal cortex (DMPFC) in interpersonal judgment (Mitchell et al., 2005b; Jenkins and Mitchell, 2010; Cooper et al., 2012). For example, Mitchell et al. (2005b) have compared neural correlates for forming impressions of other people vs. inanimate non-human objects and have found that the DMPFC is specifically engaged in processing information about other people. Another study on rapid evaluations of potential romantic partners has found that the neural activity of the DMPFC predicts the outcome of the subsequent romantic interactions (Cooper et al., 2012). Although these studies did not focus in particular on the accuracy of the preference estimation, they provide a hint that the DMPFC may play a major part in this process.

In addition, estimating the preferences of others based on intuition involves the theory of mind (ToM) that enables mentalizing (Gore and Sadler-Smith, 2011) and cognitive control, which allows the inhibition of the self-projection of one's own state (Hoch, 1988; West, 1996). For instance, if a *perceiver* (one who is required to infer the tastes of another person) is trying to guess whether a *target* (one whose tastes are predicted by the perceiver) would like to watch a Harry Potter movie, the perceiver needs to inhibit his/her own opinion from influencing the prediction (cognitive control) and to put him/herself into the target's shoes (mentalizing). Given that these processes engage DMPFC activity (Amodio and Frith, 2006; Lieberman, 2007), it is reasonable to expect that the DMPFC plays an important role in estimating preferences.

Other brain areas, such as the temporoparietal junction (TPJ) and the precuneus, have been strongly implicated in the ability to infer the mental states of others (Saxe and Kanwisher, 2003; Amodio and Frith, 2006; Mitchell, 2006; Van Overwalle and Baetens, 2009; Freeman et al., 2010b; Denny et al., 2012). The development of the ability to infer another person's mind coincides with the maturation of these structures (Sabbagh et al., 2009; Gweon et al., 2012) and, more importantly, activities in the ToM network appear to be critical for forming impressions upon seeing strangers' faces (Zaki et al., 2009; Rule et al., 2011). Taken together, these findings further imply that this network of neural structures involved in the ToM may influence the accuracy in estimating others' mental states and, therefore, may also take part in estimating the preferences of others.

The aims of the present study were to examine whether people can estimate the preferences of others based on a briefly presented facial appearance and to investigate the neural correlates of this ability. Prior to the main experiment, we ran separate sessions to select the items and targets and conducted a preliminary behavioral experiment (pretest) to confirm whether people are capable of inferring the preferences of others from facial appearances. In the main experiment, we investigated the underlying neural mechanisms. Participants were asked to estimate the preferences of targets for various items after they saw each target's facial photograph for 3 s (preference estimation task) while their brain activity was measured with functional magnetic resonance imaging (fMRI). We hypothesized that the activation of the DMPFC and other brain regions of the ToM and mentalizing network would be associated with the accuracy of the preference estimation.

## Materials and methods

### Item selection

Eighteen raters were asked to evaluate the photographs of 280 items from five categories (i.e., movies, books, bags for men and women, shoes for men and women, and foods) on preference rating scales ranging from −4 (strongly hate) to 4 (strongly like). Ten among the initial 40 items from each category were selected based on the mean and standard deviation of their preference ratings. More specifically, with the aim to minimize the overlap between the preference of the general population and the preference of a target person for a given item, we avoided the items that earned a high consensus by selecting items with large variances and intermediate levels of mean preference ratings. For movies and books whose contents were not readily recognizable from the presented photographs (i.e., movie posters and book covers), the raters were asked to answer how well they knew about each item on a 4-point scale (1, never known before; 2, know the name; 3, have not seen/read it but know the contents; 4, have seen/read it). The items rated below 3 in the knowledge score by more than half of the raters were excluded. As a result, 10 items were selected for each category and used as stimuli for the pretest. For the fMRI experiment, only two categories (i.e., movies and foods) were chosen based on the results from the pretest (see *Pretest* for details).

### Target selection

We recruited 56 undergraduate students (27 males; 22.78 ± 1.95 years) through online advertisements as targets, whose preferences were to be estimated by perceivers in the pretest and in the main experiment. In order to minimize the possibility that the perceivers had met the targets before, we ensured that the targets and the participants for the fMRI experiment had been recruited from different institutions. We took facial photographs of all 56 target candidates and filmed short self-introducing video clips starting with “Hello” in Korean. For the facial photographs, the candidates were asked to make a neutral face with a slight smile. After taking the photographs and making the films, the candidates were presented a list of items that were selected as described above and asked to evaluate them on 4-point preference scales that ranged from 1 (strongly dislike) to 4 (strongly like). All candidates were informed and agreed that the photographs and video clips would be shown to other participants in another experiment. The photos and video clips were edited into an identical frame (700 × 400 pixels); the video clips were edited to a 3-s length to contain only the part in which they say “Hello” in Korean. Four male and four female targets (eight targets in total) were chosen as the final stimuli for the pretest based on the following two criteria. First, we sorted the participants according to their similarity of appearance and selected targets who were dissimilar to each other in order to maximize the between-target variability of appearances. Second, we excluded targets who showed indistinct preferences to increase the within-target variability of the preferences. The same targets were used in the video-clip and photo conditions. For the fMRI experiment, we only included female targets in order to eliminate potential gender effects, and nine female targets were selected with the same criteria.

### Pretest

We ran a pretest before the main fMRI experiment to ensure that the participants were capable of accurately estimating another person's preference in our experimental setting. Nineteen undergraduate students (eight males, 22.79 ± 1.72 years) were recruited for the pretest. Ten participants (four males) were assigned to the photo condition in which the targets were presented in photographs and nine participants (four males) were assigned to the video clip condition in which the targets were shown in the video clips. One participant who rated all items indiscriminately as highly preferred was excluded from the analyses.

The participants were asked to make guesses about the preferences of the eight targets (target-trials) or to indicate their own preference (self-trials) for various items. In the target-trials, after a 1-s fixation, a photograph (or a video clip) of a target was shown for 3 s, and this was followed by a photograph of an item. Participants were asked to guess the target's preference for the given item within 5 s. In the self-trial, the initial letters of the participant's own name were presented instead of the facial photo. The trials were presented in a pseudorandom order. There were 90 trials for each of the five item categories: eight target-trials and one self-trial with 10 items per category. For the bags and shoes categories, 10 additional trials were added to the self-trials so that the participants could report their own preferences for the items for the opposite sex as well. As a result, the participants performed 470 trials in total. At the completion of the main task, the participants were asked to estimate the preference of the general population for each of the items.

To measure the accuracy of the preference estimations, we counted the number of trials in which the participants correctly estimated the valence of the targets' preference and then calculated the proportion of these correct trials for each category. For example, if a target's preference for a given item was 4 (strongly like) and a participant estimated it as 3 (like), then this trial was regarded as correct. In contrast, if a target's preference for a given item was 2 (dislike) and a participant estimated it as 3 (like), then this trial was considered incorrect because the participant failed to match the valence of the target's preference (i.e., in the preference ratings, 1 and 2 indicate dislike, whereas 3 and 4 indicate like, see Target Selection for details). The average accuracy scores across all of the categories were significantly above chance level (50%) [*t*_(17)_ = 8.52, *p* < 0.01, *d* = 4.13]. When we looked into each category separately, the preferences for movies, shoes, and foods were correctly estimated (all *ps* < 0.01), while the preferences for books and bags were not (all *ps* > 0.1; See Table 1).

**Table 1:** **The descriptive statistics of all conditions in the pretest and the results of the one-sample *t*-tests**.

	**Item type**						
		**Book**	**Movie**	**Shoes**	**Bag**	**Food**	**Total**
**TARGET TYPE**							
Photo	Mean	50.64	54.58	60.69	49.74	64.67	56.06
	SD	7.63	5.25	6.68	5.26	9.56	3.42
Video clip	Mean	52.59	61.35	54.75	50.11	65.79	56.92
	SD	5.07	3.46	5.75	3.83	10.69	3.08
Total	Mean	51.50	57.59	58.05	49.90	65.17	56.44
	SD	6.51	5.61	6.82	4.55	9.79	3.21
	*t*_(17)_	0.98	5.74^*^	5.01^*^	−0.09	6.57^*^	8.52^*^
	*d*	0.23	1.35	1.18	0.02	1.54	2.00

SD, standard deviation. t, t-scores from one-sample t-tests against chance level on the average accuracy scores across photo and video clip conditions.

* p < 0.05.

These results indicated that, at least in some domains, the participants could accurately estimate the preferences of others, even with very brief exposure to limited information, such as a video clip or facial appearance. However, the possibility remained that the participants might have referred to their own preferences [e.g., self-projection, as Hoch (1988) has suggested] or to the preferences of the general population instead of considering target-specific information. To test this possibility, we analyzed the correlations between the participants' preference estimations in the target-trials (estimated target preference, eTP) and their own preferences in the self-trials (self preference, SP), as well as their estimation about the preferences of the general population (general preference, GP), for each item. The correlation coefficients were converted into z-scores using Fisher's r-to-z transformation for statistical tests. The *z*-scores that were averaged across the participants were back-transformed into the r scores reported below (Michela, 1990). The average correlation between eTP and SP was *r* = 0.43, *t*_(21)_ = 9.30, *p* < 0.01, *d* = 4.05, and the average correlation between eTP and GP was *r* = 0.47, *t*_(21)_ = 11.03, *p* < 0.01, *d* = 4.81, indicating that eTP was partly influenced by SP and GP. These correlations were controlled for when we analyzed the behavioral and fMRI data in the main experiment.

Some previous studies have reported that people can make better judgments with dynamic cues rather than static cues because they contain richer information (Valenti and Costall, 1997; Balas et al., 2012). However, in our study, we did not find any significant differences between the cue type (video clip vs. photo) in the estimation accuracy, except for movie items [*t*_(17)_ = 3.13, *p* < 0.01, *d* = 1.51]. This might have been due to the relatively simple features of our video clips in which targets said a very simple word (“Hello”) and rarely made facial or body movements. In addition, the estimation accuracy did not differ when the perceiver's and target's genders were the same and when they were the opposite [*t*_(17)_ = 0.97, *ns*], but the perceivers generally estimated the preferences of the male targets better than the female targets [*t*_(17)_ = 3.66, *p* < 0.01, *d* = 1.77]. There was no significant perceiver's gender difference [*t*_(17)_ = 1.08, *ns*, d = 0.52].

## fMRI experiment

### Participants

Twenty-two college students (all females, 22.5 ± 2.28 years) participated in the fMRI experiment. We recruited only female participants to rule out potential gender effects because previous studies have reported that females are better than males at thin-slice judgments about others (Vogt and Colvin, 2003; Carney et al., 2007). All participants were right-handed and screened for a history of psychiatric or neurological diseases. This study was approved by the institutional review board of Korea University.

### Preference estimation task

**Figure 1:**
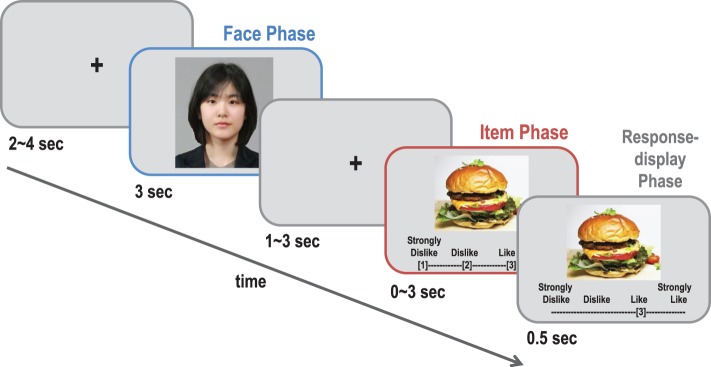
**A schematic diagram of a typical trial in the preference estimation task**. In each trial, the participants (Perceivers) were asked to guess each target's actual preference for a given item. The target's face photo (perceiver's face photo) was shown for 3 s in the target-trials (self-trials), and a photo of the item was displayed after 1~3 s. The perceivers had to estimate within 3 s how much the target liked the presented item on a 4-point scale shown below the item. Immediately after the response, their choice was shown on the screen for 0.5 s. In the self-trials, the perceivers reported their own preference for the item.

The preference estimation task for the fMRI experiment was similar to that of the pretest, except for the following details (see Figure 1). Because we found no significant difference between the photo and video clip conditions in the pretest, we used only photo cues for the fMRI experiment. A fixation phase with 1–3-s jittered fixation was added between the face phase and the item phase in order to better separate the two events in the event-related design. A 0.5-s response-display phase was added to the 3-s item phase so that the participants could see whether they pressed a response button as they intended. Unlike the pretest, the participants' own facial photographs were taken and presented in the self-trials during the face phase in order to make the visual stimuli in the self-trials comparable to those in the target-trials. Among the three categories in which the participants could estimate the preferences of others above chance level in the pretest, two categories (movies and foods) were chosen as the stimuli for the fMRI experiment. The participants performed the preference estimation task for each category separately in two scanning sessions, and the order of the two sessions was counterbalanced. Unlike the pretest, nine (all female) targets were used in the fMRI experiment. As a result, each session consisted of 10 self and 90 target-trials in total, which rendered approximately a 20-min scanning time per session. The order of the items and the targets was pseudo-randomized in order to avoid the same item or target being shown consecutively.

### Procedure

When the participants arrived at the experiment room, they were instructed about the preference estimation task and were told that the targets' actual preferences were measured in a separate session a few weeks before. To prevent the participants from responding randomly, we told the participants that they would receive additional monetary incentives depending on their performance if the accuracy level was above chance level. The participants' facial photographs were taken before they entered the scanning room. These photos were edited to the same size and resolution as those of the targets. After completing the preference estimation task inside the MRI scanner, the participants were asked to guess the preferences of the general population for every item that was shown in the scanner. The average payment for participation was approximately 30,000 KRW (≈ $30).

### fMRI data acquisition

The brain images were collected on a 3-T Siemens Trio MRI scanner (MAGNETOM Trio, A Tim System; Siemens AG, Erlangen, Germany) with a 12-channel birdcage head coil at the Korea University Brain Imaging Center. We acquired high-resolution anatomical images (*TR* = 1900 ms; *TE* = 2.52 ms; flip angle, 9 degrees; 1 × 1 × 1 mm in-plane resolution; and 256 × 256 matrix size), and then obtained functional images through gradient echo planar images (EPI) with Blood Oxygenation Level-Dependent contrast (*TR* = 2000 ms; *TE* = 30 ms; flip angle = 90 degrees; 3 × 3 × 4 mm in-plane resolution; 64 × 64 matrix size; and 33 slices with no gap).

### fMRI data analyses

The fMRI data were preprocessed and analyzed with SPM8 (Wellcome Department of Imaging Neuroscience, London, UK). The images were realigned to correct for head motion, spatially normalized to the standard Montreal Neurological Institute EPI template, and smoothed with a Gaussian kernel (6 mm full-width at half-maximum).

We constructed a general linear model for each participant including the following regressors: (1) the face phase of the self-trial, (2) the face phase of the target-trial, (3) the item phase of the self-trial along with (4) the SP rating as a parametric regressor, (5) the item phase of the target-trial along with (6) the eTP as a parametric regressor, (7) the response-display phase of the self-trial, and (8) the response-display phase of the target-trial. Additionally, six head motion regressors were included as covariates of no interest.

In order to identify the brain regions that showed significant correlations with the participants' performance on the preference estimation, we performed a regression analysis of the contrast images of the target-trials vs. the self-trials in the item phase with individual accuracy score as a covariate. Additionally, we performed a similar multiple regression analysis while controlling for the effects of SP and GP by adding the individual average correlation coefficients of the eTP with SP and GP as covariates.

Subsequently, we performed a psychophysiological interaction (PPI) analysis (Friston et al., 1997) with the peak voxel (*x* = 18, *y* = 50, *z* = 40) from the DMPFC cluster found in the multiple regression analysis as a seed region and the contrast for the main effect of the correct vs. incorrect target-trials as a psychological variable. This allowed us to identify the brain regions that showed increased functional connectivity with the DMPFC when the participants made correct estimations of the targets' preferences as compared to when they made incorrect estimations. For the PPI analysis, a design matrix was constructed to include the following three regressors: (1) the time series data from the DMPFC, (2) the psychological variable contrasting the correct and incorrect target-trials, and (3) the interaction between (1) and (2). In addition, the individual accuracy scores were regressed to the PPI between the DMPFC and other brain regions during the correct vs. incorrect target-trials. This analysis allowed us to identify the brain regions that showed stronger functional connectivity with the DMPFC in the correct than in the incorrect target-trials among the participants with higher accuracy scores.

We applied statistical significance parameters based on a peak threshold and a spatial extent threshold to correct for multiple comparisons at a level of *p* < 0.05. Using AlphaSim implemented in Analysis of Functional NeuroImages (AFNI), 1,000 Monte Carlo simulations were conducted to determine the spatial extent threshold [Parameters for AlphaSim: voxel threshold, *p* < 0.005 (uncorrected); smoothness, 8.67, 8.62, and 8.57 mm (determined by 3dFWHMx); voxel size, 2 × 2 × 2 mm]. For the multiple regression analysis with the target vs. self contrasts at the item phase regressed onto the accuracy scores, we restricted the search volumes to the brain regions involved in mentalization (80,837 mm^3^), such as the DMPFC, the TPJ, the posterior cingulate cortex (PCC)/precuneus, and the medial prefrontal cortex (MPFC), which were defined anatomically according to the Anatomical Automatic Labeling (AAL) atlas. Those brain regions were combined to create a mask of mentalization region of interest (ROI). For the multiple regression analysis with the target vs. self contrasts at the item phase that was regressed on the correlation coefficients between eTP and SP, we restricted the search volume to the VMPFC (53,330 mm^3^) that was anatomically determined based on the AAL atlas. For all of the other analyses, the whole brain volume (672,900 mm^3^) was used to determine the spatial extent threshold.

## Results

### Behavioral results

**Figure 2:**
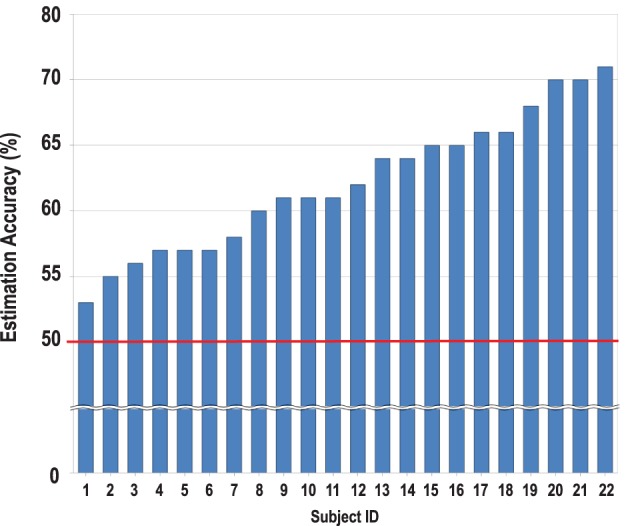
**Estimation accuracy scores of the individual perceivers**. All perceivers reached above chance level (50%, shown on the red line) [*M* = 62.18 ± 5.19, *t*_(21)_ = 11.00, *p* < 0.001].

As expected, the mean accuracy of the estimated preferences of the others for the two categories was significantly above chance level [*t*_(21)_ = 11.00, *p* < 0.001, *d* = 4.80; see Figure 2]. The mean accuracy level remained above chance level even when tested separately for movie [*t*_(21)_ = 6.68 *p* < 0.05, *d* = 2.91] and food [*t*_(21)_ = 9.61 *p* < 0.05, *d* = 4.19] items. The accuracy scores for the two categories were not significantly different from each other [*F*_(1, 19)_ = 0.35, *ns*, η^2^ = 0.02]. Thus, we combined the two categories in the subsequent analyses.

As in the pretest, we examined how the eTP was distinguished from the SP as well as the GP. The average correlation coefficient between the SP and the average of the eTP for each item was *r* = 0.52, *t*_(21)_ = 7.93, *p* < 0.01, *d* = 3.46, and the average of the correlation coefficient between the GP and the average of the eTP for each item was *r* = 0.65, *t*_(21)_ = 8.40, *p* < 0.01, *d* = 3.66. SP and GP seemed to be significantly correlated with eTP. We took this into account by statistically controlling for the effect of SP and GP in the fMRI analysis.

In addition, to verify the accuracy of the eTP even after controlling for its correlations with SP and GP, we performed a linear regression analysis on the actual target-preference (aTP) with the perceivers' eTP, SP, and GP ratings. Then, we examined the degrees to which the accuracy scores correlated with the beta coefficients of the eTP, SP, and GP ratings from the regression analysis. The accuracy scores correlated significantly with the beta coefficients of the eTP for movie items (*r* = 0.51, *p* < 0.05) but only marginally for food items (*r* = 0.40, *p* = 0.06). Beta coefficients for neither the SP nor the GP ratings showed significant correlations with accuracy scores (all *p* > 0.1). In addition, we regressed the eTP on aTP, SP, and GP. The beta coefficients of aTP were correlated significantly with the accuracy scores for food items (*r* = 0.43, *p* < 0.05) and marginally with those for movie items (*r* = 0.35, *p* = 0.11). Neither SP nor GP correlated significantly with the accuracy scores (all *p* > 0.2). In summary, although the SP and GP ratings contributed partly to the accuracy scores, the perceiver's estimations of the target's preferences seemed to be the most significant factor that accounted for the variation in the accuracy scores among the perceivers.

In addition, we examined a potential learning effect, that is, whether time or repetition had any influences on the accuracy of the estimations of the target preferences. We calculated the performances separately for each block of targets grouped by the presentation order in each perceiver and conducted a repeated measure ANOVA. This analysis yielded no significant repetition effect [*F*_(9, 387)_ = 0.61, *p* = 0.78]. We also examined if there was any potential order effect between the two separate fMRI scanning sessions in terms of estimation accuracy and again found no order effect [*F*_(1, 21)_ = 1.18, *p* = 0.29].

Finally, to examine the potential variability in terms of the readability among targets, we computed a readability score for each target by averaging the ratio of correct trials for the specific target separately for each item category (i.e., movies and foods), which indicated the degree of estimation difficulty. For example, if all perceivers correctly estimated a target's preference toward five movie items, the target's readability score for the movie category would be 5. The readability scores varied from 4.49 to 7.27 (the highest possible score was 10) for the movie category and from 4.77 to 7.90 for the food category, indicating that some targets were easier to estimate than others. Given that the correlation of the readability scores between the two categories was not significant (*r* = −0.02, *p* > 0.1), however, the variability in the readability of the targets seemed to be largely dependent on the item category rather than on the target *per se*.

### fMRI results

Our primary goal was to investigate which brain regions were involved in the process of *accurately* estimating another person's preferences with minimal information. Before addressing this question, we first explored the brain regions that engaged more when estimating the preferences of others compared to oneself. We conducted a whole brain analysis by contrasting target- vs. self-trials during the item phase. No brain region survived even at a lenient statistical threshold (*p* < 0.1, uncorrected). From the whole brain analysis contrasting the self- vs. target-trials during the item phase, we found brain regions that are known to be involved in self-reference processing, such as the MPFC (*x* = 0, *y* = 50, *z* = 6, *Z* = 5.31, corrected, *p* < 0.05), the PCC/precuneus (*x* = −10, *y* = −50, *z* = 18, *Z* = 3.58, corrected, *p* < 0.05), and the left inferior parietal cortex (*x* = −48, *y* = −46, *z* = 54, *Z* = 4.17, corrected, *p* < 0.05) (Kelley et al., 2002; Northoff et al., 2006; Sul et al., 2012), and other brain regions (See Table S1). No significant cluster was found when we contrasted the correct vs. incorrect target-trials and the incorrect vs. correct target-trials during the item phase.

### Neural correlates of the individual differences in the accuracy of estimating the preferences of others

**Figure 3:**
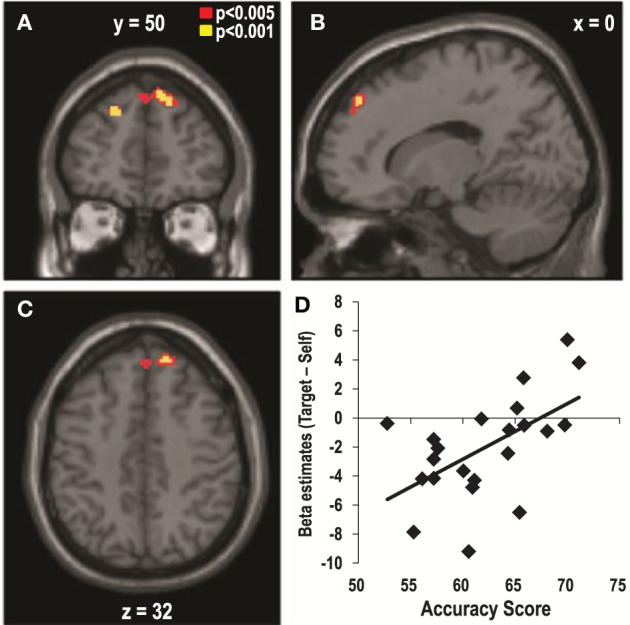
**Dorsomedial Prefrontal Cortex (DMPFC) activity predicts the accuracy of the estimations of the targets' preferences**. The DMPFC (*x* = 18, *y* = 50, *z* = 40, *Z* = 3.42, corrected, *p* < 0.05) activity that occurred in response to the target- vs. self-trials during the item phase predicted the individual variability in the accuracy of estimating the preferences of the targets. **(A)** Coronal, **(B)** Sagittal, and **(C)** Axial views of the DMPFC. **(D)** Scatter plot of the beta estimates of the DMPFC in the contrast of target- vs. self-trials as a function of accuracy scores.

As shown in Figure 2, the individual accuracy scores varied significantly across the participants, and this might have been the reason why no significant cluster was observed in the main contrasts of the previous analyses. Thus, we aimed to examine the neural correlates of the individual differences in the accuracy of estimating targets' preferences. We performed a regression analysis in which the individual contrast maps of the target- vs. self-trials during the item phase were regressed against the individual accuracy scores as a covariate. This analysis revealed that individuals with higher accuracy scores showed greater activity in the DMPFC (*x* = 18, *y* = 50, *z* = 40, *Z* = 3.42, corrected, *p* < 0.05, Figure 3, Table 2) during the evaluation of the items for the targets compared to oneself. This cluster survived even when we controlled for the effects of SP and GP by adding the correlation coefficients between eTP and SP, and eTP and GP as covariates to the same multiple regression model (*x* = 16, *y* = 52, *z* = 42, *Z* = 3.42, corrected, *p* < 0.05). We found no significant brain regions other than the DMPFC when we expanded the search volume to the whole brain. In addition, the test for the negative association between the individual accuracy scores and the target vs. self contrast did not yield any significant result.

**Table 2:** **Brain regions that exceeded the threshold determined by AlphaSim (*p* < 0.05)**.

**Brain region**	**Peak in MNI**			***Z* score**	**# of voxels**
	***x***	***y***	***z***		
**CORRELATION BETWEEN TARGET VS. SELF CONTRASTS AND ACCURACY SCORES**					
Dorsomedial prefrontal cortex (R)	18	50	40	3.42	130
**PPI WITH DMPFC DURING CORRECT VS. INCORRECT TRIALS**					
Dorsomedial prefrontal cortex (R)	18	48	42	4.37	483
	24	36	52	4.07	
	28	44	46	3.54	
Medial prefrontal cortex (L)	−4	60	2	3.83	182
Anterior cingulate cortex	0	46	−4	3.37	
Anterior cingulate cortex (R)	10	42	2	3.31	
Posterior cingulate cortex/Precuneus (R)	22	−56	40	3.78	134
	10	−58	36	3.16	
	16	−54	32	2.76	
Superior parietal lobule (R)	20	−60	70	3.52	256
	28	−72	58	3.27	
	38	−62	56	3.06	
Superior parietal lobule (L)	−26	−70	50	3.18	156
**THE EFFECT OF SP COEFFICIENTS ON TARGET VS. SELF CONTRASTS DERIVED FROM THE MULTIPLE REGRESSION ANALYSIS WITH aTP, SP, AND GP COEFFICIENTS AS REGRESSORS**					
Ventromedial prefrontal cortex (R)	20	34	−16	3.77	63
Ventral tegmental area	−6	−18	−22	3.76	146
**CORRELATION BETWEEN THE PPI WITH DMPFC AND ACCURACY SCORES**					
Posterior cingulate cortex/Precuneus (L)	−2	−44	40	3.73	344
	4	−34	42	3.25	
	0	−50	32	2.67	
Temporoparietal junction (L)	−48	−66	48	3.72	135
	−44	−68	38	2.96	
Temporoparietal junction (R)	48	−60	28	3.55	542
	58	−62	24	3.51	
	48	−66	42	3.33	
Inferior frontal gyrus (p. triangularis) (R)	40	28	18	3.29	155
	60	26	20	3.24	
	52	26	18	3.10	

MNI, Montreal Neurological Institute; R, right; L, left; PPI, psychophysiological interaction; SP, self-preference; aTP, actual target preference; GP, general preference.

### Functional connectivity between the DMPFC and other brain regions

Considering that the DMPFC is part of the neural network of mentalization along with the other ToM regions, such as the TPJ and the PCC/precuneus (Saxe and Kanwisher, 2003; Frith and Frith, 2006), we expected that the DMPFC would communicate with other structures in the network during the estimations of the targets' preferences. Specifically, we hypothesized that the communication between the DMPFC and the ToM regions would be stronger when the estimations were correct than when they were incorrect. To address this question, we performed a PPI analysis. We defined the DMPFC as a seed region and sought the brain regions that showed stronger functional connectivities with the DMPFC during the correct than the incorrect target-trials. This analysis revealed that the DMPFC (*x* = 18, *y* = 48, *z* = 42, *Z* = 4.37, corrected, *p* < 0.05), the MPFC (*x* = −4, *y* = 60, *z* = 2, *Z* = 3.83, corrected, *p* < 0.05), and the PCC/precuneus (*x* = 22, *y* = −56, *z* = 40, *Z* = 3.78, corrected, *p* < 0.05) showed stronger functional connectivity with the DMPFC when the participant's estimations for a target's preferences were correct than when they were incorrect. The other brain regions that showed significantly stronger functional connectivity with the DMPFC during the correct vs. the incorrect trials are reported in Table 2. No brain regions showed a stronger connectivity with the DMPFC during the incorrect vs. the correct target-trials.

**Figure 4:**
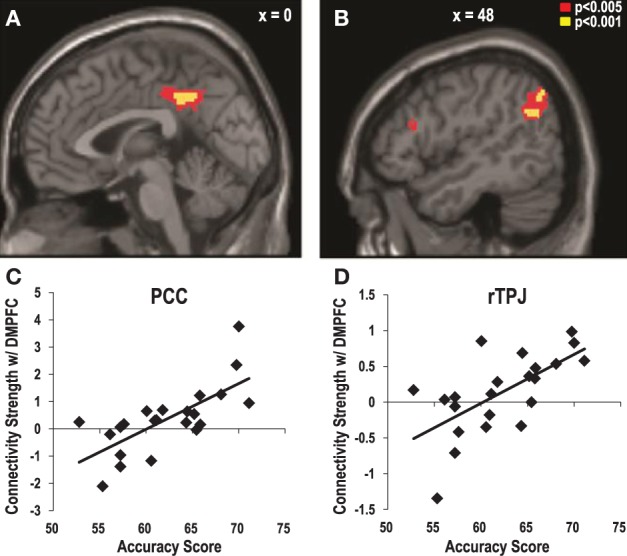
**The strength of the functional connectivity with the DMPFC associated with higher accuracy in estimating the preferences of the targets**. The psychophysiological interaction with DMPFC activity during correct vs. incorrect estimation trials that increased as a function of the individual estimation accuracy scores were observed in posterior cingulate cortex [PCC; **(A)**: *x* = −2, *y* = −44, *z* = 40, *Z* = 3.73, corrected, *p* < 0.05] and the right temporoparietal junction [TPJ; **(B)**: *x* = 48, *y* = −60, *z* = 28, *Z* = 3.55, corrected, *p* < 0.05]. Scatter plots of the connectivity strength between the DMPFC and **(C)** the PCC, and **(D)** the right TPJ as a function of the individual estimation accuracy scores.

More importantly, we also examined how the individual variations in the accuracy of the preference estimation interact with the functional connectivity between the DMPFC and the ToM regions. When the individual accuracy scores were regressed to the PPI map that was obtained from the procedure above, significant clusters were found in the PCC (*x* = −2, *y* = −44, *z* = 40, *Z* = 3.73, corrected, *p* < 0.05, Figures 4A,C) and the right TPJ (*x* = 48, *y* = −60, *z* = 28, *Z* = 3.55, corrected, *p* < 0.05, Figures 4B,D). In other words, the functional connectivity between these regions and the DMPFC became stronger among the participants with higher accuracy when they guessed correctly than when they guessed incorrectly during the target-trials. No brain region showed a negative association between the accuracy scores and its connectivity with the DMPFC.

### Neural correlates of the individual differences in utilizing other sources for estimation

**Figure 5:**
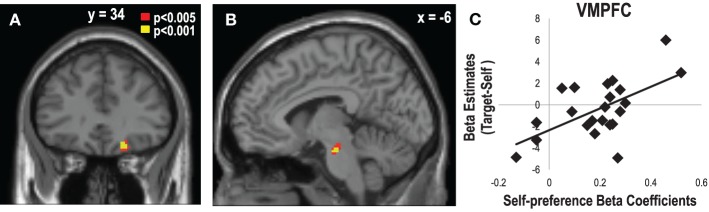
**Ventromedial Prefrontal Cortex (VMPFC) and Ventral Tegmental Area (VTA) activity associated with the impact of self-preference on the estimated target preference. (A)** The VMPFC (*x* = 20, *y* = 34, *z* = −16, *Z* = 3.77, corrected, *p* < 0.05) and **(B)** the VTA (*x* = −6, *y* = −18, *z* = −22, *Z* = 3.76, corrected, *p* = 0.06) activities during the target vs. self conditions showed positive correlations with the individual variabilities in the degree of the impact of self-preference (SP) on the estimated target preference (eTP). **(C)** Scatter plot of the beta estimates of the VMPFC in the contrast of the self- vs. target-trials as a function of the impact of SP on eTP.

In order to investigate the brain regions related to estimations based on SP or GP, we performed a multiple regression analysis on the target- vs. self-contrast maps during the item phase with the beta coefficients attained from the regression analysis that regressed the eTP on the aTP, SP, and GP in the behavioral results. This analysis allowed us to find the brain regions that are associated with the extent of the influence of SP and GP on the eTP. We found that the VMPFC (*x* = 20, *y* = 34, *z* = −16, *Z* = 3.77, corrected, *p* < 0.05) and the ventral tegmental area (*x* = −6, *y* = −18, *z* = −22, *Z* = 3.76, corrected, *p* = 0.06) activities showed significant correlations with the beta coefficients of SP (Figure 5). Consistent with the findings on the accuracy scores, the beta coefficients of aTP correlated with the activities in the DMPFC (*x* = 16, *y* = 52, *z* = 40, *Z* = 3.76, corrected, *p* < 0.05), the right inferior frontal gyrus (*x* = 50, *y* = 14, *z* = 18, *Z* = 3.57, corrected, *p* < 0.05), and the left temporal pole (*x* = 50, *y* = 8, *z* = −6, *Z* = 4.38, corrected, *p* < 0.05). There were no significant clusters correlated with the beta coefficients of GP.

## Discussion

Humans are highly social animals; the ability to estimate others' preferences in an accurate and reliable manner may be essential for successful social adaptation. In daily interactions, we often do this on the basis of minimal information. The present study demonstrated that people can estimate the preferences of others based on briefly presented subtle and non-communicative facial appearances. Participants in the present study were asked to make guesses about unfamiliar target individuals' preferences for various items after looking at their faces for 3 s. The overall accuracy of the estimations was significantly above chance level. Importantly, this remained significant even after controlling for the participants' own preferences and their beliefs about the preferences of the general population. The fMRI data revealed that higher accuracy in the preference estimations was associated with greater activity in the DMPFC when the participants guessed the targets' preferences relative to their own. This result indicate that the accurate estimation of others' preferences may require increased activity in the DMPFC. In addition, those with higher accuracy in estimating the preferences of others showed increased functional connectivity between the DMPFC and a network of ToM regions, such as the TPJ and PCC/precuneus, particularly when their estimations were correct rather than incorrect. In summary, the present study provided the first evidence that DMPFC activity may be critically related to success in estimating others' preferences and that higher accuracy may require stronger communication between the DMPFC and a network of neural structures, including the TPJ and the PCC/precuneus, which are now widely known to be involved in taking another person's perspective during mentalizing.

### Role of the DMPFC in estimating others' preferences

From both evolutionary and ontogenetic perspectives, social environments must have forced humans to develop a neural system that is specialized for estimating others' preferences. Such a system seems to require a change in mental mode, or perspective, which may critically determine the successful and accurate estimation of others' preferences. Yet, only a few neuroimaging studies have investigated the neural mechanisms underlying the accuracy of inferring personal traits from facial appearances, which has been reported to involve the amygdala (Rule et al., 2010, 2011) and the insula (Spezio et al., 2008). Unlike these studies, however, we did not find any association between these structures and the accuracy of estimating others' preferences. One possible explanation for the gap between the findings of the previous studies and the present study might be that, compared to the inference of personal traits, estimating another person's preferences may require higher-level cognitive processes, such as perspective taking and mentalization, which involve activity in the DMPFC rather than other subcortical regions. In addition, functional and anatomical evidence seem to indicate strong reciprocal connections between the DMPFC and the structures listed above (Amaral and Price, 1984; Augustine, 1996; Kim et al., 2011), suggesting that the DMPFC might be a key center in integrating signals that carry information from those subcortical structures.

The DMPFC has been considered a component of a global mentalization network (Mitchell et al., 2005a; Amodio and Frith, 2006; Frith and Frith, 2006; Mitchell, 2006; Lieberman, 2007; Schiller et al., 2009; Jenkins and Mitchell, 2010; Muscatell et al., 2012). Given that inferring information about another person involves the ToM and mentalizing ability (Gore and Sadler-Smith, 2011), it may be natural to reason that the DMPFC would play a key role in preference estimation. The DMPFC has also been implicated in various aspects of social behavior, particularly interpersonal judgments, such as forming impressions of other people or predicting the outcomes of future relationships (Walter et al., 2004; Mitchell et al., 2005b; Jenkins and Mitchell, 2010; Cooper et al., 2012), as mentioned in the Introduction, and this is more relevant to the present study. In addition, the DMPFC seems to be important for assessing the value of risky choices, especially for another person (Jung et al., 2013). Similar to the present study, Jung et al. (2013) have observed that risky decisions for others vs. oneself are related to stronger functional connectivity between the DMPFC and the structures known to be involved in mentalization, such as the TPJ and the PCC. These findings suggest that the DMPFC may be more sensitive to social evaluations rather than to one's own value assessments, as has been proposed by Cooper et al. (2010, 2012), and that the role of the DMPFC in estimating the values of another person's choices may be dependent upon integrated signals that come from a network of neural structures specialized for mentalization.

### The DMPFC as a core cognitive system

The successful estimation of others' preferences often requires cognitive control that inhibits the self-projection of one's own state (Hoch, 1988; West, 1996). That is, in order to correctly guess the preferences of others, one needs to inhibit his/her own opinion that may influence the estimation. According to a recent theoretical framework about the neural mechanisms in an attentional cognitive task, the DMPFC can be considered a core system for monitoring and modulating other attentional submodules, such as the TPJ, which are involved in stimulus-driven shifts in attention (Dosenbach et al., 2006; Corbetta et al., 2008). This theory suggests that the TPJ, a core part of the ventral attention system, acts like an efficient steering system that reorients attention from a current focus to information that is more relevant to the goal. Perhaps, the self-projection of one's own preference is a highly automatic process, and it may often be difficult to override this process, even during the estimation of others' preferences. Thus, the ventral attention system needs to be engaged to reallocate attention to more relevant external sources of information such as the appearances of others. Consistent with this hypothesis, it has been recently observed that the activation level of the mentalization network, including the right TPJ, reflects the accuracy of interpersonal inferences, based on visual information from faces, in estimating leadership competency (Rule et al., 2011) and the affective states of others (Zaki et al., 2009).

It is still debated whether modules for the ToM and for attention are colocalized or are segregated within the TPJ (Mitchell, 2008; Scholz et al., 2009). Yet, it is tempting to speculate that, when evaluating an item for another person, momentary fluctuations in the TPJ activity signaling are required to shift attention and communicate with the DMPFC in order to consider the person's facial appearance, which is the information more relevant to the task goal. As can be shown by the present findings, communication between the TPJ and the DMPFC may be critical for successful value estimations from the perspective of others. This argument is further corroborated by the fact that the strength of the functional connectivity between the DMPFC and the TPJ was stronger among participants with a higher preference estimation accuracy for correct vs. incorrect trials in the present study. It remains to be answered in future studies whether a similar network can be recruited, even when the perspective-taking aspect of the present task is substituted by a purely non-social component.

### The DMPFC and modeled choices vs. choices for others

The view of the DMPFC functioning in making choices for others has been challenged by a recent study in which DMPFC activity reflects modeled vs. executed choices rather than other vs. self choices (Nicolle et al., 2012). Despite the significance of this finding in expanding our view of the role of DMPFC in mentalization, it is important to note that the participants in the study had prior knowledge about the choices of the partners through extensive practice and, thus, the task used in the study did not require active inferences of the partners' preferences. Given that uncertainty is an inevitable key component of estimating the choices of others, the DMPFC appears to have a privileged role in inferring the preferences of others (Jenkins and Mitchell, 2010; Cooper et al., 2012; Jung et al., 2013), at least before we become fully familiar with the preferences of others. It is important to examine whether the role of the DMPFC in modeling choices changes as a function of learning the preferences of others.

### The role of the VMPFC in the estimation of others' preference

One possible way that one's estimation goes awry from the actual preferences of others may be the application of one's own preferences to the estimation process. This type of self-projection of one's own preferences appears to be a highly automatic process that is often difficult to override during the estimation of others' preferences. Interestingly, we found a large range of individual variability in the degree of self-projection during the estimation of other's preference (i.e., the beta coefficients of the SP accounting for eTP in the multiple regression analysis), and this type of individual variability was significantly predicted by the activation level of the VMPFC during the target vs. self conditions. In other words, those whose VMPFC activity increased during the estimation of others' preferences tended to project their own preferences onto the others, which could have then resulted in inaccurate estimations. A large body of literature now supports the primary role of the VMPFC in encoding subjective values critical for one's own decisions (Kim et al., 2006; Kable and Glimcher, 2007; Kim et al., 2007; Chib et al., 2009). Combining these findings with our previous account for the role of the DMPFC, it can be concluded that, for accurate and successful estimations of others' preferences, the DMPFC and TPJ need to work together and be engaged in order to control the activity of VMPFC and inhibit the intrusion of one's own preference and to reallocate the attention to more relevant external sources of information such as the appearances of others.

### Preference estimation and implicit inference about personality

What particular information from faces do people utilize for the successful estimation of others' preference? One may easily come up with a hypothesis that the preference estimation task used in the current study might be considered an applied version of the inference of the target's personality. Although the present study might require some degree of inferences about personality, the estimations about the target's preferences might not be based solely on explicit and effortful inferences about personality, especially given that the time for estimation was not long enough (~3 s) for any conscious and deliberate inferences about the target's personality. Consistent with this argument, during the debriefing, no participants reported that they tried to apply the target's inferred personality to the estimation of the target's preferences. Therefore, although it is not clear at this point what particular information from the faces the perceivers used for target-preference estimation, this type of estimation process might have been influenced by personality traits that were inferred, perhaps at an implicit level, just as some personality traits, such as extroversion and conscientiousness, can be quickly and accurately read from faces (Carney et al., 2007). These issues will need to be resolved further in future studies.

## Conclusions

The present study examined the role of the DMPFC in estimating the preferences of strangers. Consistent with the existing literature on thin slicing, the participants were able to estimate the preferences of strangers significantly above chance level, even with brief presentations of non-communicative facial appearances. Importantly, the activity in the DMPFC and close communication with the ToM and the mentalization network in the brain was found to be associated with the accuracy of the estimation. The present findings add to the literature in the rapidly growing field of decision neuroscience by providing unequivocal neural evidence for thin-slice judgments and social perception. Future studies that focus on the mechanisms underlying the individual differences in the accuracy of estimating others' preferences will also lead to fruitful outcomes in both industrial and clinical applications.

### Conflict of interest statement

The authors declare that the research was conducted in the absence of any commercial or financial relationships that could be construed as a potential conflict of interest.

## Abbreviations

SP: self-preference
eTP: estimated target preference
aTP: actual target preference
GP: general preference
TPJ: temporoparietal junction
ToM: theory of mind
DMPFC: dorsomedial prefrontal cortex
VMPFC: ventromedial prefrontal cortex
MPFC: medial prefrontal cortex
