# Supplementary material for: Dorsomedial prefrontal cortex activity predicts the accuracy in estimating others' preferences
Source: Front Hum Neurosci. 2013 Nov 26;7:686. doi: 10.3389/fnhum.2013.00686 (PMC3840299; doi:10.3389/fnhum.2013.00686)
Supplement: Supplementary file 1 [file DataSheet1.PDF]

Supplemental Data

Table S1. Brain regions exceeding the statistical threshold determined by AlphaSim (*p* <0.05)

Brain region	peak in MNI			Z	Voxels
	x	*y*	*z*		
Self > other during item phase					
Anterior Cingulate Cortex (L)	0	50	6	5.32	4547
Superior Medial Gyrus (L)	-12	50	12	4.73	
	-4	50	14	4.54	
Middle Cingulate Cortex (L)	-2	-24	40	5.28	1614
	4	-36	38	4.75	
Posterior Cingulate Cortex (L)	-4	-44	32	4.15	
Middle Frontal Gyrus (R)	22	52	28	4.68	810
Superior Medial Gyrus (R)	10	58	38	4.58	
Superior Frontal Gyrus (R)	18	58	24	3.95	
Middle Temporal Gyrus (L)	-56	-18	-20	4.27	459
	-64	-20	-20	4.09	
Inferior Temporal Gyrus (L)	-58	-20	-30	3.81	
Inferior Parietal Lobule (L)	-48	-46	54	4.17	1546
Angular Gyrus (L)	-52	-60	32	4.08	
Middle Occipital Gyrus (L)	-44	-78	32	3.71	
Inferior Frontal Gyrus (L)	-58	22	20	4.16	523
Temporal Pole (L)	-28	10	-32	3.93	
Inferior Frontal Gyrus (L)	-54	20	-2	3.44	
ParaHippocampal Gyrus (R)	30	-38	-10	4.04	396
Thalamus (R)	14	-16	4	3.92	
Insula (R)	30	-26	4	3.79	
Superior Frontal Gyrus (R)	16	28	50	3.96	215
	20	12	50	3.93	
Superior Medial Gyrus (R)	12	38	56	3.39	
Middle Occipital Gyrus (R)	38	-70	34	3.92	1079
	48	-80	24	3.49	
Angular Gyrus (R)	54	-56	32	3.66	
Inferior Temporal Gyrus (L)	-50	-54	-14	3.78	166
	-56	-58	-10	3.18	
	-50	-48	-6	2.82	
Parahippocampal Gyrus (R)	24	-4	-24	3.74	160
	28	0	-30	3.72	

	18	0	-20	3.51	
Precuneus (L)	-10	-50	18	3.58	200
	-12	-58	14	3.07	
Cuneus (L)	-10	-66	24	3.33	
